# Signals Controlling Lytic Granule Polarization at the Cytotoxic Immune Synapse

**DOI:** 10.3389/fimmu.2018.00307

**Published:** 2018-02-20

**Authors:** Anna Kabanova, Vanessa Zurli, Cosima Tatiana Baldari

**Affiliations:** ^1^Department of Life Sciences, University of Siena, Siena, Italy

**Keywords:** cytotoxic T cells, natural killer cells, immune synapse, lytic granules, cell polarity, trafficking

## Abstract

Cytotoxic immunity relies on specialized effector T cells, the cytotoxic T cells, which are endowed with specialized cytolytic machinery that permits them to induce death of their targets. Upon recognition of a target cell, cytotoxic T cells form a lytic immune synapse and by docking the microtubule-organizing center at the synaptic membrane get prepared to deliver a lethal hit of enzymes contained in lytic granules. New insights suggest that the directionality of lytic granule trafficking along the microtubules represents a fine means to tune the functional outcome of the encounter between a T cell and its target. Thus, mechanisms regulating the directionality of granule transport may have a major impact in settings characterized by evasion from the cytotoxic response, such as chronic infection and cancer. Here, we review our current knowledge on the signaling pathways implicated in the polarized trafficking at the immune synapse of cytotoxic T cells, complementing it with information on the regulation of this process in natural killer cells. Furthermore, we highlight some of the parameters which we consider critical in studying the polarized trafficking of lytic granules, including the use of freshly isolated cytotoxic T cells, and discuss some of the major open questions.

Cytotoxic T lymphocytes (CTLs) armed with lytic machinery exert a non-stop patrol of our body to identify and eliminate target cells with potentially “dangerous” phenotype. CTLs bear T cell receptors (TCRs) through which they bind antigenic peptides presented on the major histocompatibility complex (MHC) molecules of target cells. In this way, they successfully discriminate between healthy cells and those presenting non-self peptides, typically of neoplastic or microbial origin. As consequence of non-self recognition, CTLs attack and lyse malignant and infected cells. In this context, an impaired functioning of CTLs may lead to immune evasion of tumors and the insurgence of chronic infections. Hence, defining the mechanisms underlying CTL-mediated killing could provide essential insights into our understanding of immune pathology.

The immune synapse of CTLs represents a highly organized system of intercellular communication. Its assembly is initiated upon CTL recognition of a cognate target, toward which the CTL rapidly polarizes (Figure 1A). Within minutes, CTLs drastically reorganize their cytoskeleton to translocate the microtubule-organizing center (MTOC) toward the synaptic interface (1, 2). MTOC docking beneath the synapse ensures microtubule-assisted directional transport of specialized secretory lysosomes containing an arsenal of soluble cytolytic proteins, among which granzymes and perforin, and membrane-anchored effector molecules such as the Fas ligand (FasL) (3, 4). Exocytosis of secretory lysosomes at the synapse leads to a focal release of lytic enzymes into the synaptic cleft and promotes the surface exposure of FasL, thus implementing two major mechanisms of intrinsic cytotoxicity of CTLs. Killing by perforin/granzyme-dependent or FasL-dependent pathway is equally important for protective immunity and tumor surveillance since genetic deficiency for any of these molecules leads to immune pathology (5, 6). Recent evidence suggests that the two mechanisms are mediated by distinct pools of secretory lysosomes, one preferentially containing perforin and granzymes (conventionally called “lytic granules”) and the other preferentially containing FasL (4). Here, we will focus on perforin-containing lytic granules, as the biology of this vesicular compartment has so far been investigated in more detail.

**Figure 1:**
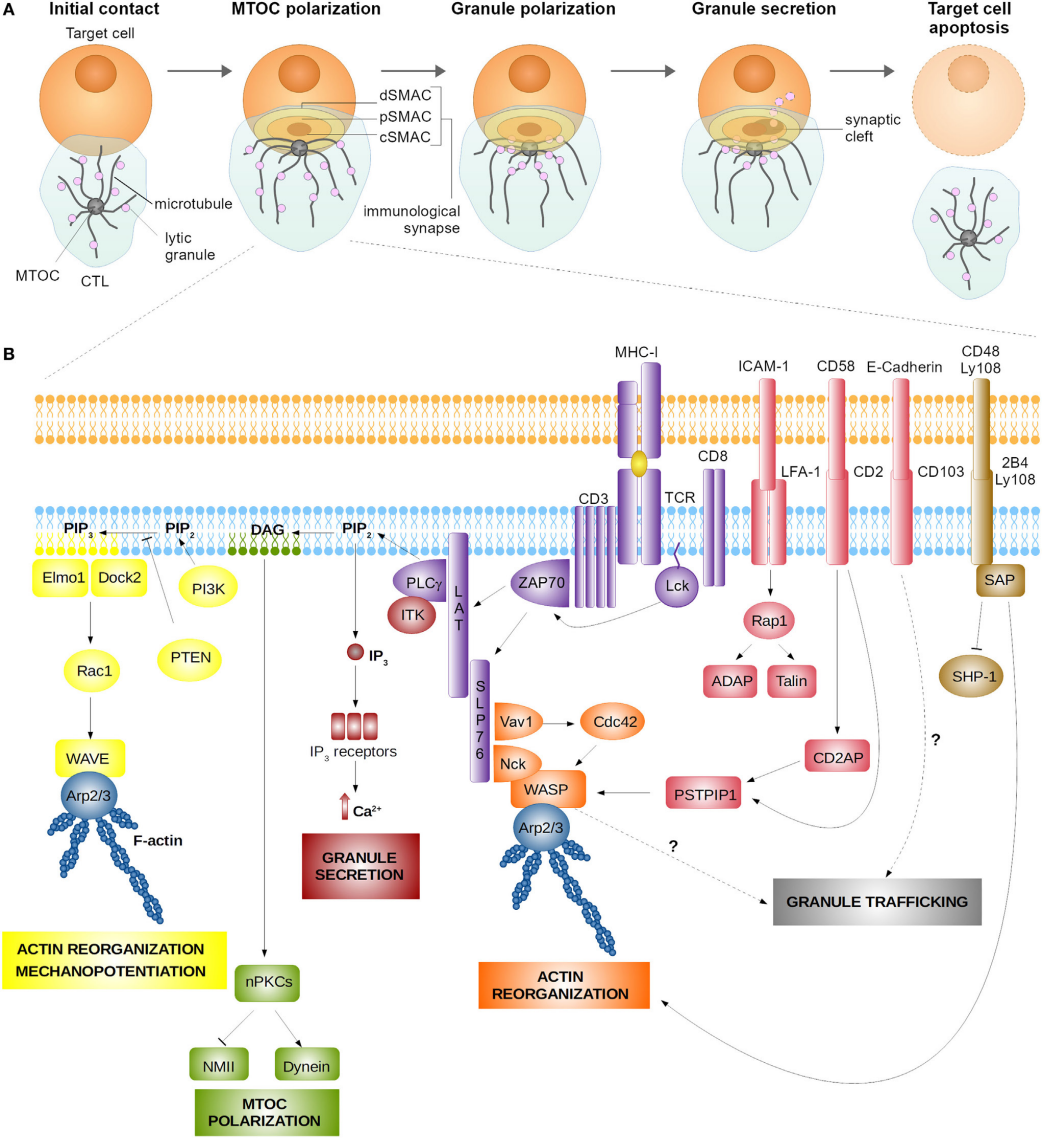
Assembly **(A)** and signaling **(B)** at the cytotoxic T lymphocyte (CTL) synapse. **(A)** Encounter between a CTL and a target cell results in the formation of the lytic immune synapse characterized by the formation of central supramolecular activation cluster (cSMAC) surrounded by the peripheral supramolecular activation cluster (pSMAC) and the distal supramolecular activation cluster (dSMAC) bearing adhesive and mechanosupportive function, respectively. Polarization of the CTL toward the synapse is characterized by microtubule-organizing center (MTOC) and lytic granule translocation toward the synaptic cleft. **(B)** Signaling at the immune synapse activates signaling modules responsible for actin cytoskeleton reorganization, which serves to stabilize the nascent synapse and enhance the cytotoxic action of CTL’s lytic molecules through a mechanopotentiation mechanism. Actin reorganization at the synapse is achieved mainly through the action of the linker for activation of T cells (LAT) signalosome and the nucleation-promotion factors Wiskott–Aldrich syndrome protein (WASP), WAVE, and Arp2/3. Synapse signaling also promotes MTOC polarization and granule secretion, which require the activity of protein kinase C isoforms (PKCs) and Ca^2+^-induced signaling pathways, respectively. Finally, engagement of adhesion receptors (LFA-1, CD2, and CD103) and signaling lymphocyte activation molecule receptors (2B4 and Ly108) aid synapse stabilization and actin reorganization. Signaling pathways implicated in granule trafficking are as yet to be established.

The polarization and exocytosis of lytic granules at the immune synapse are driven by local signaling, which triggers two major polarization events, namely (i) the polarization and docking of the MTOC at the synapse and (ii) the polarization of lytic granules toward the docked MTOC, followed by granule exocytosis or “degranulation,” which results in the focal release of granule contents. Notably, at variance with the mechanisms regulating MTOC translocation, the signaling events that regulate lytic granule polarization remain as yet to be fully characterized. Understanding how granule convergence at the synapse is regulated could shed substantial insight into the mechanisms driving CTL dysfunction.

Here, we present an overview of the mechanisms that orchestrate the polarized transport of lytic granules in CTLs and incorporate some relevant information on the signaling of natural killer (NK) cells for a comparative analysis of polarizing signals in these two types of cytotoxic lymphocytes. We also discuss recent experimental evidence describing dysfunctional non-polarized immune synapses. Finally, we comment on the parameters of experimental setups that we consider important for the study of polarized trafficking in CTLs and highlight the major open questions concerning its orchestration.

## Directionality of Granule Secretion in CTLs is Dictated by the TCR and Costimulatory Signals

Many of the signaling pathways contributing to the assembly of the CTL synapse have been dissected (Table 1). Loss-of-function studies, interestingly, allowed us to establish that CTL polarization is controlled at two levels: at the level of early polarization events regulating MTOC recruitment and at the level of late polarization and fusion events regulating granule convergence toward the MTOC and degranulation into the synaptic cleft. Regulation of late polarization events in CTLs could have a critical impact on their functioning, as it has recently emerged that uncoupling of granule movement from MTOC polarization during synapse assembly may lead to a dysfunctional non-polarized pattern of degranulation (7). This type of degranulation (also referred to as “non-directional” or “non-lytic”) has been described for NK cells as well (8, 9). As such, non-polarized degranulation may represent a means to finely tune the functioning of cytotoxic lymphocytes. Understanding molecular differences between polarized versus non-polarized CTL synapses in terms of synaptic signaling, corresponding properties of the target cells and the CTLs themselves could be highly instrumental for understanding the mechanisms regulating cytotoxic killing.

**Table 1:** Loss-of-function analysis of signaling molecules that orchestrate CTL synapse assembly.

Defective protein	Cytotoxicity	Conjugate formation	MTOC polarization	Granule polarization	Degranulation	Method of interference
**Proximal signaling molecules of the TCR cascade**						
Lck	−	+	No docking	−	−	Genetic KO (23)
Fyn	−	+	−	−	−	Genetic KO (23)
ZAP-70	−	+	−	−	−	Inhibitor (22)
LAT	Decrease	+	Decrease	Decrease	N/a	Genetic KO (42)
Itk	−	+	+	+	−	Genetic KO (40)

**Integrins and cytoskeleton**						
LFA1	Decrease	N/a	N/a	Decrease	+	Blocking Fab (10)
	Decrease	Decrease	Decrease	N/a	N/a	Blocking Ab and inhibitory action of galectins (52)
CD103	−	N/a	N/a	Decrease	Decrease	siRNA KD of E-cadherin on target cells (44)
	−	Decrease	N/a	Stimulated by E-cadherin coated beads	Blocked by siRNA E-cadherin KD	siRNA KD of E-cadherin on target cells (48, 49)
WASP	Decrease	N/a	+	Decrease	+	Genetic KO (84)

**Calcium signaling**						
OraI1	−	+	+	+	−	Pharmacological inhibition of store-operated Ca^2+^ entry (36)

**Costimulation**						
SAP	Decrease	Decrease	−	N/a	N/a	Genetic KO (105)

**Molecules co-localizing with lytic granules**						
PKCδ	−	+	+	−	−	Genetic KO (135, 136)

*N/a, not assessed; +, preservation of function; −, complete loss-of-function; KO, knockout; KD, knockdown; CTL, cytotoxic T lymphocyte; MTOC, microtubule-organizing left; TCR, T cell receptor; WASP, Wiskott–Aldrich syndrome protein; LAT, linker for activation of T cells; E-cadherin, epithelial cadherin-1; Itk, interleukin-2-inducible T cell kinase; PKC, protein kinase C isoform*.

Signaling initiated upon target recognition by the CTL is dictated by the TCR, which recognizes antigenic peptides presented by MHC class I molecules on the target and by costimulatory signals triggered through the engagement of CTL surface receptors by various ligands expressed on the target. Signaling studies on planar lipid bilayers coated with defined molecular ligands allowed to establish that essentially only two types of molecules may be triggered on a CTL to induce synapse formation (10). These are the TCR and the integrin LFA-1 that functions as an adhesion molecule stabilizing the contact between the two cells. However, when CTL interact with a cellular target, TCR/integrin signaling is further integrated by various costimulatory and inhibitory signals that contribute to CTL regulation.

An important notion that has emerged from the first molecular studies of CD4^+^ T cell synapse formation is that the TCR and some of the costimulation/inhibitory molecules are spatially segregated during synapse assembly. This stands true also for the mature immune synapse of CTLs. Upon the initiation of CTL synapse assembly, the TCRs and proximal signaling molecules of the TCR pathway concentrate into the central area of the synapse [known as the central supramolecular activation cluster (cSMAC)], while LFA-1 and other adhesion molecules segregate into a ring which surrounds the cSMAC [peripheral supramolecular activation cluster (pSMAC)] (10–12). The pSMAC is itself enclosed by a dense ring of filamentous actin (F-actin), which defines the distal SMAC (dSMAC) that forms the outer boundary of the immune synapse (13). The three concentric regions, which are formed upon CTL synapse maturation, carry out distinct functions. The cSMAC serves as a hub for proximal signaling and active secretion at the synapse (11), the pSMAC initiates and stabilizes the adhesion between CTLs and its target (10), while the actin cytoskeleton at the dSMAC exerts mechanical force across the synapse to potentiate and direct cytotoxic killing (14).

Interestingly, the formation of a mature immune synapse with signaling molecules arranged in the SMAC pattern does not constitute a pre-requisite for successful killing by CTL. It has been shown that as few as two to three peptide/MHC complexes on the surface of the target can trigger cytotoxicity (15, 16). In such setting, the polarization of the CTL lytic machinery toward the synapse can occur in the absence of the large-scale molecular rearrangements characteristic of the SMACs (16–18). Based on these findings, such CTL synapses have been proposed to be referred to as “lytic,” as opposed to the “stimulatory” mature SMAC-bearing synapses (17). Thus, the functional importance of SMAC formation for CTL cytotoxicity remains an open question.

In this review, we will summarize the current knowledge on the TCR and costimulatory signaling pathways in CTLs and discuss their contribution to the regulation of polarized synaptic secretion (Figure 1B). For the sake of simplicity, we will structure this review from the cSMAC, pSMAC, and dSMAC perspective, although it should be underscored that the distribution of signaling molecules in T cell synapses is generally highly mobile, as is the SMAC architecture (19, 20).

## The CTL cSMAC is the Area of MTOC Docking and Degranulation

Proximal TCR signaling at the cSMAC is initiated by the two Src family kinases Lck and Fyn (21). Lck activity is required for further propagation of signaling through ζ-chain-associated protein kinase of 70 kDa (ZAP-70) (22). Activation of proximal TCR signaling molecules is of crucial importance for the induction of CTL polarization, since the activity of Lck, Fyn, and ZAP-70 are all essential for promoting MTOC polarization to the lytic synapse (22–25).

Activated ZAP-70 phosphorylates another important molecule of the TCR pathway, the linker for activation of T cells (LAT), which recruits numerous signaling effectors to form a multiprotein complex termed the LAT signalosome (26). The LAT signalosome includes several important signaling molecules, among which the SH2 domain-containing leukocyte protein of 76 kDa (SLP76), phospholipase Cγ1 (PLCγ1), interleukin-2-inducible T cell kinase (Itk), and the Rho family GTPase exchange factor Vav1. These signaling mediators couple TCR activation to intracellular processes important for CTL functioning, such as MTOC polarization, Ca^2+^ signaling, and cytoskeletal reorganization (21).

SLP76 mediates the activation of PLCγ1, which exerts two major functions at the lytic synapse of CTLs, namely the induction of MTOC polarization and Ca^2+^ influx (21). At the cSMAC, PLCγ1 catalyzes the hydrolysis of phosphatidylinositol 4,5-bisphosphate (PIP_2_) leading to the formation of the lipid second messengers diacylglycerol (DAG) and inositol 1,4,5-trisphosphate (IP_3_). The local DAG gradient promotes the synaptic recruitment of protein kinase C (PKC) isoforms, namely novel PKCs including PKCθ, PKCε, and PKCη, which participate in the proximal signaling inducing MTOC polarization (1, 27, 28). Also, the member of the atypical PKC family, PKCζ, participates in the signaling pathway driving polarization at the T cell synapse (29), thus suggesting that the cascade of signaling events transduced through PKC isoforms are crucial for the establishment and maintenance of T cell polarity (30). Activity of these PKCs and the concomitant synaptic accumulation of dynein, the minus-end-directed microtubule motor, are essential to promote MTOC translocation toward the synapse, which occurs within minutes after the initiation of synapse assembly (1). This delineates the TCR–PLCγ1–DAG–PKC axis as the signaling module responsible for MTOC polarization toward the CTL synapse. Interestingly, also the actin cytoskeleton contributes to MTOC polarization. Indeed, it has been reported that PKCs regulate the actin-based motor non-muscle myosin II, which acts in synergy with dynein to move the MTOC toward the synapse (31).

Once translocation to the subsynaptic area has occurred, the MTOC docks at the plasma membrane, which promotes the focused microtubule-directed transport and exocytosis of lytic granules directly into the synaptic cleft (11, 32). Two molecular motors appear to be involved in the process of granule polarization toward the MTOC, which is thought to occur in parallel with the MTOC translocation to the synapse. First, on their way toward the MTOC, granules are transported through a dynein-dependent mechanism in the direction of microtubule minus-ends (3, 33). Then, during the final phase of synapse assembly, kinesin-1 and the small GTPase Rab27a mediate the plus end-directed movement of granules to bring them from the MTOC into close proximity of the CTL plasma membrane, thus allowing for granule fusion and secretion (34).

Interestingly, under certain experimental conditions MTOC polarization toward the synapse does not represent a requirement for polarized granule secretion and cytotoxicity. Granule movement and release toward the target (35), or even toward multiple targets recognized by the CTL simultaneously (18), may precede MTOC polarization. As the movement of granules and MTOC is uncoupled in these settings, this suggests that granules could reach the synapse through a plus end-directed movement. These results provide additional support to the ability of granules to move bidirectionally, raising the question as to which are the signaling pathways that drive granule mobilization in general and tune the directionality of their transport specifically.

The second major outcome of TCR-triggered PLCγ1 activity is the induction of Ca^2+^ influx into the CTL through the activity of PLCγ1-generated IP_3_ (36). IP_3_ binds to IP_3_ receptors in the endoplasmic reticulum (ER) membrane and activates Ca^2+^ release from the ER into the cytoplasm (37). The initial increase in the cytoplasmic Ca^2+^ triggers the store-operated entry of extracellular Ca^2+^ into the T cell through the channels formed by a complex of the ER calcium sensors STIM1 and STIM2 and the Ca^2+^ release-activated calcium channel ORAI1 at the plasma membrane (38). Ca^2+^ mobilization from the extracellular space is an essential requirement for CTL degranulation as highlighted by a selective defect in lytic granule exocytosis upon pharmacological inhibition of store-operated Ca^2+^ entry in CTLs (36). Hence, the modulation of IP_3_ levels achieved through regulation of PLCγ1 represents a means to control CTL functioning. Activity of Itk, a component of the LAT signalosome, is important for full activation of PLCγ1 (39). Consistently, a deficiency in Itk leads to a defect in CTL degranulation (40), thus confirming a fundamental role of the Itk-PLCγ1-IP_3_ axis in regulating Ca^2+^ signaling and lytic granule exocytosis in CTLs.

Besides modulating the production of IP_3_, Itk appears to generate some additional signals important for CTL degranulation since the Ca^2+^ ionophore-induced Ca^2+^ influx is not sufficient to correct Itk deficiency (40). The components of this signaling pathway are as yet unknown. Another layer of complexity in the picture of Ca^2+^-dependent molecular events that occur during synapse assembly is the fact that neither is IP_3_ the only second messenger regulating Ca^2+^ signaling, nor is the ER the only Ca^2+^ store in CTLs. Nicotinic acid adenine dinucleotide phosphate (NAADP), the most potent Ca^2+^-releasing second messenger known, is also produced following TCR stimulation and crucially contributes to the signaling pathway operating during CTL degranulation (41). NAADP mobilizes Ca^2+^ selectively from acidic stores, such as the lytic granules themselves, and, when added to cultured CTLs *in vitro*, is able to trigger their degranulation (41). Thus, Ca^2+^ signaling in CTLs is mediated synergistically by IP_3_ and NAADP. However, the whole spectrum of signaling molecules contributing to this branch of CTL signaling has yet to be fully defined.

It is noteworthy that Ca^2+^ signaling in CTLs impacts only on the process of lytic granule exocytosis, without influencing either MTOC or lytic granule polarization (36, 40), which demonstrates a clear diversification of the signaling pathways orchestrating CTL cytotoxicity. Surprisingly little is known about the signaling pathways that trigger granule trafficking along the microtubules and determine the directionality of their transport, although few signaling parameters have emerged as important regulatory factors. One is the strength of TCR stimulation, as TCR triggering with low affinity ligands leads to an impairment in lytic granule polarization toward the MTOC, which has been associated with defective Lck activation (24). The kinetics of intracellular Ca^2+^ flux has also been implicated in the regulation of lytic granule polarization. Indeed, a second major class of cytotoxic T cells, CD4^+^ CTLs, show slower granule polarization at the synapse and slower degranulation when compared to conventional CD8^+^ CTLs (12). This peculiar phenotype has been associated with diminished Ca^2+^ influx in CD4^+^ CTLs compared to CD8^+^ CTLs.

Finally, our laboratory recently demonstrated that the assembly of a functional CTL synapse strictly depends on properties of the target. For instance, we showed that primary human B cells can instruct CTLs to degranulate non-directionally (7). Early signaling TCR events (e.g., ZAP-70 phosphorylation), intracellular Ca^2+^ influx, and MTOC polarization in such non-polarized synapses were normal, while the activation of LAT was selectively impaired. LAT has been previously implicated in CTL cytotoxicity (42). However, a complete loss of LAT in CTLs was shown to affect multiple steps of synapse formation, including its stability, and led to an impairment of both MTOC and granule polarization. Therefore, it is still not clear whether LAT acts as a global regulator of CTLs functioning or might be implicated in the fine regulation of CTL polarity, as direct evidence for LAT function in lytic granule trafficking is still missing.

Thus, we still lack information about the TCR signaling originating at the cSMAC that determines the directionality of granule trafficking, although rapid Ca^2+^ kinetics and the activation of LAT may serve as indicators of the successful granule polarization at the CTL synapse.

## The pSMAC Supports CTL Adhesion and Costimulation

Adhesion molecules at the CTL synapse play a dual role: they physically stabilize the nascent contact between CTL and a target and trigger important signaling pathways in the CTLs that contribute to synapse maturation. Several adhesion molecules promoting cytotoxic killing have been identified, including LFA-1 (also known as CD11a/CD18 or αL/β2 integrin) (10), CD2 (also known as LFA-2) (43), and CD103 (also known as αEβ7 integrin) (44). The ligands for those integrins on the target cells are the intracellular adhesion molecule 1 (ICAM-1), lymphocyte function-associated antigen 3 [LFA-3 or CD58; CD48 in mice (45)], and epithelial cadherin-1 (E-cadherin), respectively. Interestingly, the properties of target cells, i.e., which integrin ligands they express, define the signaling requirements for cytotoxic killing. For instance, killing of cells of hematopoietic origin requires the interaction of LFA-1 with ICAM-1 (46) and of CD2 with CD58 (47), while cytotoxic lysis of epithelial cells requires CD103/E-cadherin interaction (44, 48, 49). Of note, among these molecules, only LFA-1 was described as clearly segregating to the pSMAC upon synapse formation (10, 43). This property has never been investigated for CD103, while CD2 behaves differently when studied in different experimental systems. In studies on Jurkat T cells interacting with stimulatory lipid bilayers, CD2 is closely associated with the TCR at early time points of the immune synapse formation, but eventually segregates to the pSMAC surrounding the TCR-containing cSMAC (43). At variance, when studied on murine T cells activated by cellular targets, CD2 remains in close association with the cSMAC structure, diffusing toward the synapse periphery only minimally (50).

The best-studied integrin for T cells is LFA-1. Its engagement on CTLs has been shown to facilitate CTL killing by enhancing CTL adhesion to the target and stabilizing the nascent synapse (10, 51, 52). Shortly after the onset of synapse assembly, LFA-1 establishes high-affinity interactions with ICAM-1 on the target cell, as the result of a conformational change in its extracellular domain triggered by the “inside-out” signaling of engaged TCRs (53), and translocates toward the pSMAC (10). Inside-out signaling operates through the small GTPase Rap1, the scaffold protein RIAM, the cytoskeletal protein talin, and local membrane PIP_2_, which promote the recruitment of LFA-1 to the immune synapse and help it to acquire a stable high-affinity conformation [reviewed in Ref. (53)]. In turn, the outside-in costimulatory signaling of engaged LFA-1 involves its association with the activated kinases Lck and ZAP-70 and signal spreading through Vav1 and SLP76 (54). Interestingly, both talin and Vav1, which actively participate in integrin signaling, are also implicated in the rearrangement of the actin cytoskeleton (discussed below), thus functionally linking the pSMAC to the adjacent dSMAC, the main mechanosupportive structure of the CTL synapse.

Along with LFA-1, CTL costimulation through CD2 appears to be highly important for CTL functioning (47, 55). Of note, the TCR and CD2 pathways are significantly interdependent as CD2 signaling proceeds through the cytoplasmic domain of the TCR-CD3ζ subunit, at least as shown in Jurkat T cells (56, 57). CD2 is also capable of transducing the signal *via* the Fc epsilon RI gamma subunit (58), thus explaining why also cells lacking surface TCR, such as double-negative thymocytes and NK cells, can be activated *via* CD2 stimulation (59). Similar to LFA-1, TCR activation leads to an increase in the avidity of CD2 interaction with CD58 (60), suggesting that inside-out signaling of the TCR also plays a role in CD2 activation. Both Fyn activation (61) and the adaptors LAT (43, 62) and the Wiskott–Aldrich syndrome protein (WASP) (63) have been implicated in outside-in signaling by CD2, at least in CD4 T cells. Fyn activation by CD2 has been linked to the activation of the signaling axis composed of PLCγ1, Vav1, PKC theta, docking protein (Dok), focal adhesion kinase (FAK), and protein tyrosine kinase 2 (Pyk2) (61). Interestingly, FAK and Pyk2 participate in the maintenance of focal adhesions, which are multimolecular complexes linking surface integrins to the actin cytoskeleton [reviewed in Ref. (64)], while WASP is a key molecule participating in the reorganization of actin cytoskeleton in T cells (65). Their activation is also triggered following TCR engagement, thus CD2 signaling could strengthen the activation of the actin-remodeling signaling branch, functionally connecting the pSMAC with the dSMAC.

Finally, CD103 appears to be directly implicated in the regulation of CTL polarity. CD103 engagement by E-cadherin on epithelial cells promotes polarization of lytic granules at the CTL synapse through PLCγ1 activity (44, 48, 49), albeit the precise signaling pathway mediating this process has not been characterized yet.

Hence, the pSMAC of CTL synapse represents an important structure with adhesive and costimulatory function that critically contributes to the reorganization of CTL cytoskeleton. Interestingly, while signaling at the pSMAC is not able to autonomously promote CTL polarization, totally depending in this on the TCR engagement, in NK cells LFA-1 signaling is *per se* sufficient to promote MTOC and granule polarization at the immune synapse (8, 66). In these cells, the signaling axis of LFA-1 signaling is centered on an integrin-linked kinase (ILK)–Pyk2–paxillin core and the ILK-controlled cdc42-Par6 pathway, which regulates polarity in other cell types (67). Thus, an in-depth analysis of adhesion receptor signaling in CTLs has the potential to broaden our understanding of the signals orchestrating the directionality of granule trafficking.

## The dSMAC: An Area of Extensive Actin Cytoskeleton Remodeling at the CTL Synapse

To support granule secretion toward the target, CTLs rely on a robust remodeling of their cytoskeleton. The dynamics of F-actin, which ultimately accumulates at the dSMAC forming an F-actin ring, plays a central role in synapse maintenance and regulation of granule secretion. Notably, F-actin remodeling in CTLs has two major outcomes. First, it controls the direction and timing of lytic granule release, acting as a dynamic physical barrier for secretion (2, 68). Second, it exerts a strong force across the synapse, enhancing perforin activity at the plasma membrane of the target cells and thus potentiating cytotoxicity (69). The importance of the actin cytoskeleton for CTL functioning is confirmed by the fact that F-actin disruption or impaired functioning of cytoskeleton regulators such as Vav1 and WASP affect CTL killing (13, 70–72).

Initially, an extensive actin polymerization at the synapse leads to the formation of a dense cortical actin layer, aiding CTL spreading over the surface of the target. By 1 min after contact, cortical F-actin starts a retrograde movement toward the periphery of the synapse that concludes with the formation of an F-actin ring delineating the future dSMAC (2). The continuous centrifugal movement of actin within the dSMAC has been shown to strengthen the adhesive forces mediated by integrins/adhesion molecules at the pSMAC (73). Finally, F-actin retrograde flow leads to a depletion of cortical actin from the center of the synapse generating an actin hypodense region that will control the access of lytic granules to the plasma membrane (2). Subsequently, the cortical cytoskeleton barrier recovers across the synapse as granule secretion is completed, with the full cycle of events being completed within approximately 30 min (68).

T cell receptor signaling is the main trigger responsible for actin cytoskeleton remodeling at the lytic synapse. The LAT-SLP76 complex plays a central role in this process by activating second messenger pathways and by directly recruiting actin regulators (13, 74, 75). To achieve this, phosphorylated SLP76 physically associates with two important signaling molecules, the phosphoinositide 3-kinase (PI3K) (76) and Vav1 (75, 77). PI3K is responsible for the production of phosphatidylinositol (3,4-5)-phosphate (PIP_3_) from PIP_2_, which has been identified as a key event promoting the spatial segregation of F-actin to the dSMAC ring (13). Local PIP_3_ further promotes the recruitment of the guanine nucleotide exchange factors Dock2-Elmo1 that associate with the Rho family GTPase Rac1 (78). Phosphorylated Vav1, instead, associates with another Rho family GTPase, namely Cdc42 (75). Rac1 and Cdc42 are responsible for the activation of two major actin nucleation-promoting factors: the WASP family verprolin-homologous protein (WAVE) and WASP itself, respectively (79, 80). Thus, the LAT-SLP76 complex appears to be the initiating point and the central signaling hub orchestrating the process of actin reorganization at the synapse. Interestingly, loss-of-function studies have demonstrated that even though the PI3K-PIP_3_-Dock2 axis is crucial for the efficiency of CTL killing, it does not regulate the intracellular trafficking of lytic granules (13). Instead, PI3K-PIP_3_-Dock2 activity is related to the generation of mechanical force exerted by the T cell across the synapse, which translates into a significant mechanopotentiation and improved perforin pore formation (69).

Activated by Cdc42 and Rac1, WASP and WAVE control F-actin assembly by promoting the nucleation of actin filaments (65, 80). In support of the importance of F-actin and WASP in the maintenance of an effective immune synapse, it has been reported that T cells from Wiskott–Aldrich syndrome patients, who are WASP-deficient, as well as those from WASP knockout mice, form unstable synapses due to cytoskeletal defects (72, 81–83). Also, WASP deficiency in human CTLs has been associated with a reduced cytotoxicity against tumor B cell lines specifically caused by incomplete lytic granule polarization toward the target (84). In line with this, studies on NK cells have further confirmed that WASP and WASP-interacting protein regulate the polarization of granules toward the synapse (85–87). These findings support the notion that WASP may be involved in the signaling pathway that regulates granule trafficking in CTLs.

Notably, even though actin dynamics are also important for the formation of the NK immune synapse, a crucial difference exists between the synapses of NK cells and CTLs in the cortical cytoskeleton distribution. At variance with CTLs, in NK cells, lytic granule secretion occurs through a dense F-actin meshwork containing granule-sized clearances (88, 89). Also, NK cell granules are constitutively associated with the motor myosin IIA that promotes their interaction with the F-actin-rich cell cortex at the synaptic membrane and assists their final transit toward the synaptic cleft (90). Thus, considering both similarities and differences between CTLs and NK cells, a deeper investigation on the actin organization and function in relation to the granule trafficking process could prove to be very important in establishing the whole spectrum of factors regulating cytotoxicity.

The key role of F-actin in the formation of lytic immune synapses has been also studied in the context of cancer evasion from immune surveillance. In particular, it has been reported that CTLs from patients with B cell chronic lymphocytic leukemia establish defective synapses characterized by an impaired polarization of F-actin (91). Moreover, we have shown that non-lytic synapses between non-directionally degranulating CTLs and B cells are characterized by a decreased polymerization of F-actin at the interface between the two cells (7). These results suggest an important role for F-actin in late polarization events at the synapse between CTLs and target cells.

Taken together, actin dynamics at the immune synapse may clearly impact the process of granule polarization. Interestingly, the key F-actin regulator WASP also participates in CD2 signaling (see “pSMAC” section). Thus, WASP could be recruited to the synapse by two mechanisms: through LAT-SLP76 signaling (92) and through its association with the actin adapter PSTPIP that interacts with CD2 both directly and through the CD2AP adapter (63). Taking into account the major role of adhesive and costimulatory molecules for CTL cytotoxicity (44, 46, 47), it would be of great interest to study the involvement of CD2 signaling, LAT signalosome assembly, and WASP activity in F-actin dynamics in both lytic and non-lytic synapses.

## Costimulatory Signaling Lymphocyte Activation Molecule (SLAM) Receptors at the CTL Synapse

The SLAM family members have been extensively studied in CTL functioning due to their involvement in the X-linked lymphoproliferative (XLP) syndrome (93, 94). XLP disease is a rare genetic disorder characterized by abnormal responses to the Epstein–Barr virus infection due to a deficiency in the SLAM-associated protein adaptor SAP, which modulates signaling downstream of SLAM receptors in CTLs (95–97). The SLAM receptors have pleotropic functions in the immune system (98), with SLAM, 2B4, NTB-A, Ly108, and CRACC receptors playing an important role in CTL and NK cell cytotoxicity (93, 99–104). With the exception of 2B4, which interacts with CD48, all SLAM receptors establish homotypic interactions, being expressed both on CTLs and their targets.

Although SLAM receptors are important for CTL-mediated cytotoxicity (103, 104), there are only few studies focused on SLAM receptor signaling at the lytic synapse and its impact on CTL polarization events. Among these, two reports have deepened our understanding of the role of SLAM receptors in the context of CTL and B cell target encounter. These studies clearly highlighted that coupling of SAP with activated 2B4 and Ly108 is essential to trigger positive signals enhancing TCR signaling and to promote cytoskeleton reorganization and correct CTL polarization (93, 105), while preventing the recruitment of the inhibitory phosphatase SHP-1 at the synapse (105). In NK cells, SAP couples activated 2B4 to the kinase Fyn, which in turn activates Vav1, while blocking the inhibitory phosphatase SHIP-1 from binding to 2B4 (106). Another signaling adaptor of SLAM receptors, EAT-2, controls instead MTOC and granule polarization to the NK cell synapse by linking SLAM family receptors to phospholipase Cγ, calcium mobilization, and Erk kinase (107).

Interestingly, studies on human and murine NK cells have demonstrated that LAT is constitutively associated with 2B4 in glycolipid-enriched membrane microdomains and has a critical role in SLAM-mediated cytotoxicity (108–110). Considering that the activation of the LAT signalosome has a key role in actin polymerization at the dSMAC (74, 75) and may be involved in the signaling controlling granule polarization toward B cell targets (7), it would be important to further our knowledge on the connection between SLAM receptors and polarization signaling in CTLs.

## Considerations on the Experimental Setups for Studying Polarized Granule Trafficking in CTLs

### Use of *In Vitro* Expanded CTLs versus Freshly Isolated CTLs

Lytic synapse assembly is typically studied using CTLs expanded *in vitro* after their isolation from human or mouse donors. CTL expansion in this case is usually required due to a relatively low percentage of effector cytolytic cells among the total pool of CD8^+^ T cells [in healthy human donors 10–15% (7)]. An *in vitro* expansion of the heterogeneous CD8^+^ T cell population is also convenient because it allows for the enrichment of highly cytolytic CTLs with unique antigen specificity. A caveat of this experimental setup is that the expansion of primary T cells typically requires their maintenance in medium supplemented with high quantities of mitogenic cytokines, e.g., interleukin-2 (IL-2). It has become evident that culturing CTLs in IL-2-supplemented medium may alter signaling requirements for CTL killing when compared to freshly isolated effector CTLs. In particular, this knowledge emerged from studies on CTLs from patients and mice with genetic mutations that affect CTL functioning. For instance, function-disrupting mutations in Munc18-4, which regulates lytic granule fusion with the plasma membrane, abolish cytotoxicity in freshly isolated CTLs due to a loss of their ability to degranulate (111). However, culturing these dysfunctional CTLs for 9 days with IL-2 leads to a compensation of the genetic defect (111). Likewise, a defective functioning of CTLs from mice with Itk deficiency is restored upon 6–7-day exposure to IL-2 (40). A similar functional recovery has been observed for CTLs with mutated dysfunctional syntaxin 11 (112), WASP (84, 113), or LYST (114). An effect of IL-2 culturing on killing properties has also been observed in NK cells. IL-2 pulsed NK cells from healthy donors are able to lyse ICAM-1-expressing targets, whereas the sole LFA-1 triggering is not sufficient to trigger cytotoxic killing by freshly isolated NK cells (8). IL-2 culturing also rescues cytotoxicity in otherwise dysfunctional NK cells from patients with familial hemophagocytic lymphohistiocytosis (112, 115). Hence, *in vitro* expansion of primary cytotoxic lymphocytes appears to have a profound effect on their properties, likely by altering expression levels of proteins implicated in CTL synapse assembly, as suggested (111).

We observed that an *in vitro* expansion influences the ability of human CTLs to polarize lytic granules at the synapse (7). As discussed earlier, primary human B cells can instruct freshly isolated CTLs to degranulate in a non-polarized dysfunctional fashion. However, CTL blasts expanded *in vitro* from their freshly isolated counterparts are able to polarize lytic granules toward B cell targets, which correlates with the restoration of their killing potential (7). This observation suggests that freshly isolated CTLs and CTL blasts have different signaling requirements for granule polarization. Therefore, studies on polarized trafficking in CTLs may potentially benefit from including freshly isolated CTLs in the experimental setup. The use of freshly isolated CTLs could also be relevant for high-throughput screenings and high-content studies that until now have been largely performed on *in vitro* expanded cells (116, 117).

### Experimental Setups to Study Cytotoxic Function of Freshly Isolated CTLs

Human CTLs endowed with high cytotoxic potential could be defined as CD8^+^ CD57^+^ CD27^−^ CD28^−^ perforin/granzyme-rich population in the peripheral blood of donors (118–120). These cells represent a polyclonal population with mixed TCR specificities expanded *in vivo* by natural stimuli. Such CTLs can be isolated from the total CD8^+^ T cell pool using flow cytometry sorting [e.g., to purify CD45RA^−^/CD45RA^+^ CCR7^−^ CD28^−^ CD27^−^ effector-type populations (121)] or using two alternative magnetic bead-based enrichment protocols: (i) depletion of CD27^+^ cells (7, 118) or (ii) magnetic bead-based depletion of CD28^+^ CD45RO^+^ cells (119). Effector CTLs isolated using either of these methods would be, however, of mixed specificities, with T cell clones responding to individual antigenic peptides present at too low frequency for an efficient experimental setup. To overcome this limitation, historically two systems of polyclonal CTL stimulation, aimed at promoting synapse formation with target cells, have been developed.

The first system relies on the use of bacterial superantigens (SAgs) that are able to cross-link the MHC class II molecules on target cells and the TCRs on CTLs (bearing a particular TCR V β chain, specific for every type of SAg) (122). In this setting, SAg may bind to multiple individual TCRs, thus ensuring an elevated number of CTLs that can respond to SAg stimulation (even higher when a combination of SAg is used). Although SAg is recognized in the context of MHC class II molecules, they are potent inducers of CTL cytotoxicity through an MHC class I/CD8-independent mechanism (123). The peculiarity of SAg system consists in the induction of a non-canonical TCR signaling that depends on the activation of heterotrimeric GTP-binding protein Gα_11_ and PLCβ, instead of Lck and PLCγ1, at least as shown for CD4^+^ T cells (124, 125). Nevertheless, SAg stimulation induces the formation of a “classical” immune synapse between MHC class II-bearing targets and CTLs, either freshly isolated or expanded *in vitro*, and is therefore widely used (7, 35, 52).

The second system of polyclonal stimulation commonly employed to work with human CTLs involves the use of Fc receptor-bearing target cells (e.g., P815 murine mastocytoma cells) loaded with CD3-specific monoclonal antibody. Antibody-coated targets are recognized by the CTLs through direct binding of the TCR to the antibody, thus promoting cytotoxic killing in the so-called “redirected” assay (3, 119). Of note, TCR cross-linking by CD3-specific antibody also induces the activation of Gα proteins, which draws parallels between CTL activation in the SAg-dependent system and in the CD3-based redirected assay (124, 126).

Although both systems appear to work well with freshly isolated CTLs, the use of MHC class I-peptide system is obviously more physiologically relevant as it may allow to assess the functioning of CTLs with natural ligands of different affinity and specificity. The solution to overcome the limitation of rare individual clones in the total CTL population came with the development of systems to clone and express recombinant TCR receptors, derived from naturally occurring CTL clones, in polyclonal freshly isolated CTLs (127). For instance, the TCRα and TCRβ chains of the TCR complex can be transfected into CTLs in combination, or cloned as a single multicistronic construct separated by a cleavable peptide (T2A, P2A, etc.) (128, 129), thus allowing for the simultaneous expression of the two proteins that form a functional TCR complex (130, 131). Recombinant TCR constructs can be introduced into primary T cells using a retrovirus/lentivirus-based gene transfer, or using mRNA electroporation, which is becoming a widespread technique due to a high efficiency and relative simplicity of the protocol compared to virus-based systems (127, 132, 133). Another important aspect to be taken into consideration is that the virus-based systems frequently require T cell activation prior to viral transduction, while mRNA electroporation can be performed with high efficiency even on resting T cells. Thus, mRNA electroporation with recombinant TCR constructs may represent a convenient tool to study immune synapse formation in freshly isolated CTLs stimulated with natural peptide ligands.

## Conclusion

Directionality of lytic granule transport along microtubules represent fine means to regulate immune synapse assembly in CTLs and NK cells, thus representing a basic factor in immune regulation. However, the mechanisms defining granule ability to travel bidirectionally along microtubules and, most importantly, the signaling pathways regulating this process are largely elusive. To address this question of outstanding scientific importance, we believe that future research on CTLs should focus on some specific issues. First, it would be of high relevance to identify surface receptors that trigger the signaling pathways driving granule translocation toward the MTOC. So far, it emerges that TCR triggering alone could not be sufficient to induce granule polarization, at least in freshly isolated human CTLs (7), suggesting that full CTL polarization is likely achieved through specific costimulation. Identification of surface receptors responsible for such costimulation would be central to dissecting the mechanisms regulating cytotoxic killing. Among possible candidates, CD103 could be one of such costimulatory receptors since its engagement can induce granule polarization (48). However, CD103 expression is restricted only to a minor subset of CTLs (44), suggesting that other CTL surface receptors are likely involved in this process.

Second, lysosomes have emerged as important signaling hubs implicated in the regulation of multiple cellular processes including metabolic signaling and autophagy (134). Along these lines, it would be highly interesting to determine the whole set of signaling molecules, e.g., kinases and adaptors, that associate with lytic granules. So far, only PKCδ was found to colocalize with lytic granules and appears to regulate their polarization and exocytosis in a kinase-dependent manner (135, 136), albeit its substrates and precise molecular mechanism of action have as yet not been clarified. Other interesting molecules associated with lytic granules could be identified possibly through the highly sensitive proteomics approach, which has never been performed on freshly isolated CTLs but has been done for the granules of *in vitro* expanded CTL blasts (4, 137). We anticipate that this approach would be highly instrumental for the dissection of granule biology, in particular, and of lysosomal biology in T cells in general.

Finally, it is becoming widely accepted that stimulation with cytokines is capable of reorganizing the molecular and signaling network of granule trafficking and secretion in cytotoxic lymphocytes (113). However, the effect of CTL exposure to immune stimuli that may influence their functioning has not been addressed in a systematic and broad manner, for example, through genomics or transcriptomics studies. Not only cytokines may be considered in this context but also chemokines which have a well-known function of T cell costimulation through triggering of the respective chemokine receptors (138). Thus, the field of study of lysosomal (granule) trafficking in CTLs is open wide to new contributions.

## Author Contributions

AK and VZ wrote the initial draft for the manuscript which was then revised and contributed by CB.

## Conflict of Interest Statement

The authors declare that the research was conducted in the absence of any commercial or financial relationships that could be construed as a potential conflict of interest. The reviewer AA declared a past collaboration with one of the authors CB to the handling editor.
